# Renal Cell Carcinoma Health Disparities in Stage and Mortality among American Indians/Alaska Natives and Hispanic Americans: Comparison of National Cancer Database and Arizona Cancer Registry Data

**DOI:** 10.3390/cancers13050990

**Published:** 2021-02-27

**Authors:** Celina I. Valencia, Samer Asmar, Chiu-Hsieh Hsu, Francine C. Gachupin, Ava C. Wong, Juan Chipollini, Benjamin R. Lee, Ken Batai

**Affiliations:** 1 Cancer Prevention and Control Program, University of Arizona Cancer Center, Tucson, AZ 85724, USA; celina@arizona.edu; 2 Department of Epidemiology and Biostatistics, University of Arizona, Tucson, AZ 85724, USA; pchhsu@arizona.edu; 3 Department of Surgery, University of Arizona, Tucson, AZ 85724, USA; Samerasmar94@surgery.arizona.edu; 4 Department of Family and Community Medicine, University of Arizona, Tucson, AZ 85724, USA; fcgachupin@arizona.edu; 5 Department of Urology, University of Arizona, Tucson, AZ 85724, USA; awong@email.arizona.edu (A.C.W.); jchipollini@arizona.edu (J.C.); brlee@arizona.edu (B.R.L.)

**Keywords:** cancer health disparities, socioeconomic status, geospatial, neighborhood effects, Latino health paradox

## Abstract

**Simple Summary:**

This study assessed renal cell carcinoma disparities in American Indians/Alaska Natives and Hispanic Americans using the National Cancer Database and the Arizona Cancer Registry, focusing on advanced-stage and mortality. Renal cell carcinoma disparities in American Indians/Alaska Natives have been partially explained by neighborhood socioeconomic factors and residence (rural or urban) pattern, but not in Hispanic Americans. Greater health disparities in renal cell carcinoma stage and mortality for Hispanic Americans and renal cell carcinoma mortality for American Indians/Alaska Natives were observed at the Arizona state level compared to national levels.

**Abstract:**

Renal cell carcinoma (RCC) is one of the top 10 cancers in the United States. This study assessed RCC health disparities in American Indians/Alaska Natives (AIs/ANs) and Hispanic Americans (HAs) focusing on advanced-stage and mortality. RCC patients’ data were obtained from the National Cancer Database (NCDB) and Arizona Cancer Registry (ACR). Logistic and Cox regression analyses were performed to ascertain the effect of race/ethnicity on stage and mortality, adjusting for neighborhood socioeconomic factors, rural/urban residence pattern, and other factors. In both data sets, AIs/ANs had significantly increased odds of advanced-stage RCC in the unadjusted model, but not in adjusted models. Mexican Americans had higher odds of advanced-stage compared to non-Hispanic Whites in NCDB (OR 1.22, 95% CI: 1.11–1.35) and ACR (OR 2.02, 95% CI: 1.58–2.58), even after adjusting for neighborhood characteristics. AIs/ANs did not show increased mortality risk in NCDB after adjusting for neighborhood characteristics, while the association remained significant in ACR (HR 1.33, 95% CI: 1.03–1.72). The great risk of all-cause and RCC-specific mortality was observed in U.S.-born Mexican Americans in Arizona (HR 3.21, 95% CI: 2.61–3.98 and sub-distribution HR 2.79, 95% CI: 2.05–3.81). RCC disparities in AIs/ANs is partially explained by neighborhood factors, but not in HAs.

## 1. Introduction

Racial/ethnic groups in the United States (U.S.) have different incidences of kidney cancer and mortality rates. Non-Hispanic Blacks (NHBs) and American Indians and Alaska Natives (AIs/ANs) have higher kidney cancer incidence and mortality rates than non-Hispanic Whites (NHWs) [1]. Globally kidney cancer incidence rates have increased over the last several decades as a result of improved imaging and diagnosis [2]. The greatest increase in kidney cancer incidence rates have been observed in Latin American countries [3,4]. The incidence rates reported for Hispanic Americans (HAs), Latinos/Latinas living in the U.S., are much higher than rates reported in Latin America. In U.S.-Mexico border states, HAs have higher kidney cancer incidence rates than NHWs [5], and the U.S.-born HAs in California and Texas have higher kidney cancer mortality rates than NHWs [6].

Previous studies of kidney cancer health disparities have focused on differences between NHBs and NHWs [7,8,9,10]. Kidney cancer trends and the patterns of disparities in AIs/ANs and HAs at national- and regional-levels are not well studied. AIs/ANs and HAs have socioeconomic disadvantages as well as structural, geographic, and individual barriers to healthcare [11,12,13,14], but underlying causes of the disparities have not been understood in these populations. Previous kidney cancer studies had limited geographic scope in their analyses or focused primarily on epidemiological data without an in-depth analysis of the complex dimensions of socioeconomic and geographic factors that may be driving kidney cancer disparities [5,6,15,16,17]. The cancer disparities literature has broadly established the role of structural determinants of cancer outcomes, such as persistent poverty [18] and neighborhood effects [19]. The intersection of structural barriers and neighborhood characteristics in the form of redlining [20] and segregation [19,21] has begun to emerge as a salient consideration of cancer outcomes across the cancer continuum. This body of work provides a strong empirical basis for this study.

The state of Arizona, located in the Southwest region of the U.S., provides a unique opportunity to examine RCC disparities in HA and AI/AN communities. As a U.S./Mexico border state, Arizona has a HA population of 31.7% in metropolitan and rural areas [22]. The 2018 U.S. Census estimates that 13.6% of Arizona’s population are foreign-born individuals with Mexico as the country of origin for 54.8% of this immigrant population. Arizona has the third highest population of federally recognized tribal members at 9.79% of the state residents being AIs/ANs. Phoenix, the state’s capital, has the highest percentage of urban AI/AN residents in the U.S. [23]. Additionally, Arizona has three of the largest federally recognized tribal reservations within the state. These geographic areas, the U.S./Mexico border region and the tribal reservations, are also categorized as medically underserved areas by the Arizona Department of Health Services [24]. The tribal reservations and the U.S./Mexico border HA communities are located in rural areas requiring HAs and AIs/HAs to travel long-distances to major medical centers in metropolitan and urban areas which places substantial constraints on HAs and AIs/ANs seeking to obtain cancer screening, surveillance, and treatment. Cancer prevention and control programs often funded by state or Center for Disease Control and Prevention generally focus on common cancer types, but to date there are no programs specifically addressing kidney cancer prevention and treatment.

The primary goal of this study was to assess disparities in renal cell carcinoma (RCC), the most common type of kidney cancer, at the state (Arizona) and national level. This study focused on advanced-stage (stage III/IV) RCC, indicative of delayed diagnosis and/or treatment, and mortality in AIs/ANs and HAs, previously understudied groups who often live in medically underserved areas. While various factors, including genetics, obesity, comorbidity, and environmental and occupational exposures, may increase RCC risk in these populations, this study assessed if neighborhood socioeconomic factors and residence pattern (urban or rural) account for RCC health disparities in AIs/ANs and HAs. Early detection and timely surgical treatment (evidenced by early-stage RCC) is likely to reduce kidney cancer health disparities. The potential causes for late diagnosis and delayed treatment initiation such as neighborhood socioeconomic factors, rural residence, and healthcare access among AIs/ANs and HAs need to be understood in order to reduce kidney cancer health disparities.

## 2. Materials and Methods

To assess the pattern of RCC disparities nationally, demographic and clinicopathologic information of RCC patients who were diagnosed between 2004 and 2016 was obtained from National Cancer Database (NCDB). The NCDB is jointly sponsored by the American College of Surgeons and the American Cancer Society [25,26]. This is a clinical oncology database containing data collected from more than 1500 Commission on Cancer-accredited facilities. Since 1996, all Commission on Cancer-accredited programs, which constitute nearly 25% of all hospitals in the U.S. have been required to report cancers that are diagnosed and treated at their facilities to the NCDB. The Commission on Cancer-accredited hospitals tend to be higher volume centers than non-accredited centers, and cases from accredited hospitals account for 70% of the cancer diagnosed annually in the U.S. The NCDB is standardized data of patient demographics, tumor characteristics, surgery performed, primary treatment, cancer recurrence, and survival.

For Arizona state-level analysis of RCC disparities, data on RCC cases between 2007 and 2016 were obtained from the Arizona Cancer Registry (ACR). The ACR is a population-based surveillance system that collects, manages, and analyzes information pertaining to the incidence and mortality of people diagnosed with cancer. The registry captures and describes cancer in Arizona using a variety of information from patients including site of origin, gender, age, race, ethnicity, geographic area, and year of diagnosis. The ACR provides a platform to monitor cancer incidence, conduct epidemiologic studies, and ultimately contribute to quality improvement in detection, diagnosis, and treatment of patients.

This study focused solely on RCC and other subtypes of kidney cancer were excluded. This is a secondary data analysis using de-identified patient information and was exempt from University of Arizona Institutional Review Board approval.

This study only included cases with known race/ethnicity from the NCDB. Heterogeneity among HAs was assessed by their origin (Mexico, Puerto Rico, Cuba, South or Central America, and Dominican Republic). For ACR analysis, NHWs, AI/ANs, and HAs were included. Other racial/ethnic groups and cases with unknown race/ethnicity were excluded due to the small number of cases reported in the registry. Among HAs in Arizona, associations with advanced-stage RCC and mortality were assessed for Mexican Americans aggregately and separately for U.S.-born and foreign-born Mexican Americans.

Advanced-stage RCC includes stage III and IV based on American Joint Committee on Cancer staging system using pathologic stage group and clinical stage group when pathologic stage was not reported. For the NCDB data, effect of neighborhood characteristics and residence pattern on stage and mortality was assessed using educational attainment (proportion of adults who did not graduate from high school) and median household income based on zip code from the 2012 American Community Survey data (2008–2012) and the 2013 U.S. Department of Agriculture Economic Research Service Rural-Urban Continuum Codes. Patients’ access to healthcare was assessed using “great circle” distance (distance in miles between zip code of patient’s residence and the hospital that reported the case), insurance type, and facility type. For state-level comparison on stage and mortality, U.S. Census-tract data on socioeconomic measures for high school graduation, poverty rate, and unemployment rate were used in conjunction with the ACR data. The 2013 Rural-Urban Continuum Codes were integrated into the ACR analysis to provide an additional dimension of neighborhood characteristics.

Logistic regression was performed to ascertain the effect of race/ethnicity and Hispanic origins on advanced-stage RCC adjusting for patients’ neighborhood socioeconomic characteristics, residence patterns, healthcare access, and other relevant factors. For the analysis of NCDB data, the initial adjusted model included age category, gender, RCC histologic subtype, facility type (community cancer program, comprehensive community cancer program, academic/research program, or integrated network cancer program), insurance type, Charlson/Deyo Score (comorbidity score), and year of diagnosis (Adjusted Model 1). All the variables included in the adjusted model 1 were associated with advanced-stage RCC (at least one category in the variables with *p* < 0.05). To assess effects of neighborhood and residence characteristics, high school education (≥21%, 13–20.9%, 7–12.9%, or <7%), median household income (<$38,000, $38,000–$47,999, $48,000–$62,999, or ≥$63,000), Rural-Urban Continuum Codes (metro counties, urban counties, or rural counties), and great circle distance (10 miles increment) were added (Adjusted Model 2). Similarly for ACR, the initial model adjusted for age category, gender, RCC subtypes, and diagnosis year (Adjusted Model 1). Then, percent unemployment (<5%. 5–10%, and ≥10%), percent poverty (<10%, 10–20%, and ≥20%), percent high school graduation rate (≥90%, ≥70, <91%, and <70%), and Rural-Urban Continuum Codes 2013 (1 or 2 vs. 3–7) were added. Percent high school graduation rate was not included in the final model (Adjusted Model 2). Separate analyses for AIs/ANs and Mexican Americans were performed in each dataset to identify specific factor associated with advanced-stage RCC (Supplementary Methods).

Cox regression analysis were performed to obtain the effect of race/ethnicity on all-cause mortality. Unadjusted model included all RCC histologic subtypes for both NCDB and ACR data even if histologic subtype was not specified. A similar procedure described above was used to determine final adjusted regression models excluding unspecified RCC subtype. The initial model in the NCDB analysis adjusted for age category, gender, RCC histologic subtype, stage (I/II vs. III/IV), grade (1 and 2 vs. 3 and 4), facility type, insurance type, Charlson/Deyo Score, and year of diagnosis (Adjusted Model 1). Neighborhood characteristics (median income and high school graduation), Rural-Urban Continuum Codes, and great circle distanced were added to the model. The Rural-Urban Continuum Codes not associated with all-cause mortality were excluded in the Adjusted Model 2, resulting in the final model adjusting for age category, gender, RCC histologic subtype, stage, grade, facility type, insurance type, great circle distance, neighborhood characteristics (median income and high school graduation), Charlson/Deyo Score, and year of diagnosis.

For analysis of ACR data, the initial model adjusted for age category, gender, marital status, RCC subtypes, stage grade, and diagnosis year. Next, percent high school graduation rate, percent unemployment, percent poverty rate, and Rural-Urban Continuum Codes were added to the model. The Rural-Urban Continuum Codes were not associated with all-cause mortality, so the final model included age category, gender, marital status, RCC subtypes, stage, grade, diagnosis year, percent high school graduation rate, percent unemployment, and percent poverty rate. In ACR data, sub-distribution Cox proportional hazards regression was performed to evaluate time to death due to RCC accounting for competing risks (i.e., death due to other causes). The same adjusted models described for all-cause mortality were used.

## 3. Results

### 3.1. RCC Case Characteristics

A total of 405,073 cases from the NCDB were included in the analysis (Table S1). The largest racial/ethnic group in the study sample was NHWs (74.6%; *n* = 302,230), followed by HAs (12.2%; *n* = 49,308), NHBs (11.2%; *n* = 45,334), Asian Americans (1.6%; *n* = 6390), and AIs/ANs (0.4%; *n* = 1811). Among HAs, Mexican Americans (Mexican descent/Chicano) were the largest group (*n* = 3745). As we previously reported, racial/ethnic minority groups had a younger age of diagnosis [27]. There were more male cases (*n* = 252,616) than female cases (*n* = 152,457). AIs/ANs (42.6%), NHBs (40.9%), and HAs (39.6%) had a higher proportion of female cases than NHWs (36.9%). With regards to the tumor grade, 65.1% of the patients had grade 1 or 2 RCC distributed similarly across all groups. Advanced-stage RCC was more prevalent in AIs/ANs (31.5%, *p* < 0.001) and HAs (28.6%, *p* = 0.02) compared to NHWs (27.7%). Among HAs, Mexicans/Chicanos had the highest prevalence of advanced-stage RCC (34.1%).

A total of 9337 cases were identified in the ACR (Table S2). Mexican Americans were the largest HA subgroup (*n* = 739). AI/AN (58.9) and HA (59.3) patients had a younger age at diagnosis than NHW (64.3). Overall, there were more male RCC cases (*n* = 5978) than female cases (*n* = 3359). HA (39.5%) females had a higher proportion of RCC diagnosis than NHW females (35.0%). AIs/ANs were more likely to have lower grade RCC than NHWs (70.6% vs. 64.9%, *p* = 0.045). On the other hand, high-grade RCC was more common in Mexican Americans than NHWs (40.2% vs. 35.1%, *p* = 0.052). Compared to NHWs, advanced-stage-RCC was more prevalent in AIs/ANs (31.5% vs. 26.4%, *p* = 0.006) and Mexican Americans (47.2% vs. 26.4%, *p* < 0.001), particularly in U.S.-born Mexican Americans (49.1%).

### 3.2. Advanced-Stage RCC in AIs/ANs and HAs

At both the national- and Arizona state-level, AIs/ANs had increased odds of advanced-stage RCC compared to NHWs in unadjusted model, but the association was no longer statistically significant after adjusting for neighborhood characteristics (Table 1). Overall, HAs did not have increased odds of advanced-stage RCC nationally or in Arizona. However, compared to NHWs, Mexican Americans had 22% higher odds of advanced-stage RCC in NCDB (OR 1.22, 95% CI: 1.11–1.35) and 2-fold increased odds of advanced stage RCC in Arizona (OR 2.02, 95% CI: 1.58–2.58), even after adjusting for neighborhood characteristics. Moreover, in Arizona, both U.S.-born Mexican Americans and foreign-born Mexican Americans had similarly higher odds of advanced-stage RCC compared to NHWs (OR 2.27, 95% CI: 1.62–3.18 and OR 2.05, 95% CI: 1.39–3.03 respectively), even after adjusting for neighborhood characteristics.

Stratified analysis was performed for AIs/ANs and Mexican Americans to identify specific neighborhood socioeconomic characteristics and healthcare access factors associated with advanced-stage RCC in these populations. In NCDB, older age increased the odds of advanced-stage in both AIs/ANs and Mexican Americans (Tables S3 and S4). Greater distance to healthcare facility increased the odds of advanced-stage RCC in AIs/ANs. Mexican Americans who did not have insurance (OR 1.49, 95% C.I.: 1.06–2.10) and were treated at academic/research institutions (OR 1.26, 95% C.I.: 1.02–1.56) had higher odds of advanced-stage RCC. Mexican Americans living in neighborhoods with higher high school graduation rates had reduced odds of advanced-stage RCC. In Arizona, diagnosis in recent years were associated with increased odds of advanced-stage RCC in both AIs/ANs and Mexican Americans (Tables S5 and S6). Among AIs/ANs, low high school graduation rates were associated with increased odds of advanced-stage RCC (OR 2.73, 95% CI: 1.04–7.17).

### 3.3. Risk of all-Cause and RCC Specific Mortality in AIs/ANs and HAs

In NCDB dataset when compared to NHWs, AIs/ANs showed increased all-cause mortality risk in unadjusted model, but the association was not statistically significant in adjusted models (Table 2). Overall, HA ethnicity was associated with reduced risk of all-cause mortality in both adjusted models compared to NHWs (HR 0.93, 95% CI: 0.90–0.96 and HR 0.91, 95% CI: 0.88–0.94). The association between HA ethnicity and all-cause mortality was stronger among early-stage than advanced stage RCC (Table S7). Among HAs, Puerto Ricans and HAs of South or Central American origin there was a reduced risk of mortality (HR 0.78, 95% CI: 0.61–0.99 and HR 0.72, 95% CI: 0.59–0.88, respectively). Puerto Ricans with early-stage RCC had the greatest reduced risk (HR 0.56, 95%: 0.39–0.81), but the association was not statistically significant among Puerto Ricans with advanced-stage RCC.

Contrary to NCDB, AIs/ANs in Arizona showed increased all-cause mortality risk in both unadjusted and adjusted model with 33% increased risk in adjusted model compared to NHWs (HR 1.33, 95% CI: 1.03–1.72) (Table 3). Overall, HA ethnicity was not associated with all-cause mortality after adjusting for neighborhood characteristics, but Mexican Americans had significantly higher risk of mortality compared to NHWs in both unadjusted and adjusted models (HR 2.46, 95% CI: 2.23–2.72 and HR 2.30, 95% CI: 1.91–2.94). U.S.-born Mexican Americans had the greatest risk of all-cause mortality (HR 3.21, 95% CI: 2.61–3.98). Foreign-born Mexican Americans also showed a significantly increased risk (HR 1.50, 95% CI: 1.09–2.06), but the association was not significant among foreign-born Mexican Americans with early-stage RCC (Table S8).

In ACR data, there was a total of 1762 patients who died. There were 750 RCC attributable deaths and 1012 deaths from other causes. Significantly increased risk of RCC-specific mortality was observed only for Mexican Americans compared to NHWs (sub-distribution HR 1.76, 95% CI: 1.60–2.38). The risk was greater for Mexican Americans with early-stage RCC than late-stage RCC (Table S9). U.S.-born Mexican Americans had the greatest risk of RCC-specific mortality (sub-distribution HR 2.79, 95% CI: 2.05–3.81), while there was no association for foreign-born Mexican Americans.

## 4. Discussion

The primary goal of this study was to assess RCC health disparities in two previously understudied racial/ethnic minority groups that often live in medically underserved areas. The study demonstrates that RCC health disparities exist in AIs/ANs and HAs. Both AIs/ANs and HAs, particularly Mexican Americans, were more likely than NHWs to be diagnosed or undergo surgical treatment with more advanced-stage RCC. When neighborhood characteristics were included in the regression models, they attenuated the associations for AIs/ANs but not for HAs. AIs/ANs had an increased risk of all-cause mortality at the state-level in Arizona. Nationally, HAs had reduced all-cause mortality risk, but in Arizona, Mexican Americans, particularly U.S.-born Mexican Americans, showed an increased risk for both all-cause and RCC-specific mortality even after adjusting for neighborhood characteristics.

High kidney cancer burden in AI/ANs have been previously recognized [28]. Historically, kidney cancer has been ranked as one of the top five cancers for incidence and top 10 for mortality in AIs/ANs. National kidney cancer incidence rates were 66% higher in AI/AN males and 85% higher in AI/AN females than in NHW males and females between 2010 and 2015 [15,29]. AIs/ANs also have a greater risk of dying from kidney cancer than any other racial/ethnic groups [1,28,29]. At the national level, AI/AN kidney cancer mortality rate is elevated compared to NHWs, but the mortality rates vary regionally, exceeding more than 2-fold increased mortality rate in some areas [28,29]. The Southwest region of the U.S. has the greatest disparities supporting the higher mortality risk in AIs/ANs compared to NHWs found at the state level in this study.

Obesity, diabetes, hypertension, smoking, and low physical activities are some associated RCC risk factors that are common in AIs/ANs, and the prevalence of these risk factors vary regionally [30]. These risk factors alone may not account for the AI/AN disparities of kidney cancer mortality. This RCC focused study shows that distance to healthcare facility was positively associated with advanced-stage RCC in NCDB data, and when neighborhood socioeconomic factors were included in the model, they attenuated the associations with advanced-stage RCC and mortality in AIs/ANs. Impact of healthcare access and socioeconomic factors on RCC treatment is still unknown in AIs/ANs, and understanding this relationship is likely key for reducing RCC disparities.

HA is an aggregate term for heterogeneous subgroups from various origins with different cultures, health behaviors, and genetic admixtures. These subgroups also have historically different healthcare access across the U.S. Mexican Americans and HAs from Central and South America are less likely to utilize healthcare services [13]. HAs are more likely to be uninsured in the U.S. both pre- and post-implementation of the Affordable Care Act [14,31]. Mortality rates also vary among HA subgroups for many cancer types [32,33,34,35]. Similar to the findings from the current study, Martinez-Tyson et al. [33] found that among HA subgroups Mexican Americans have a higher kidney cancer mortality rate than other HA subgroups.

Moreover, findings from the NCDB showed HAs had an increased odds of advanced-stage RCC, and Mexican Americans without health insurance had increased odds of advanced-stage RCC. However, HAs tandemly had a reduced risk of all-cause mortality. These findings align with the Hispanic Health Paradox. The Hispanic Health Paradox stipulates that HAs have lower mortality despite a higher epidemiological risk profile than NHWs and other racial/ethnic minority groups [36,37]. On the other hand, the findings from the ACR indicated that Mexican Americans were at an increased odds of advanced-stage RCC and increased risk of RCC-specific and all-cause mortality suggesting that the Hispanic Health Paradox does not apply well to Mexican Americans in Arizona. Hispanic Health Paradox is generally more pronounced in foreign-born individuals with a diminishing epidemiological presence in subsequent generations of U.S.-born HAs [38]. Compared to foreign-born HAs, U.S.-born HAs have higher mortality rates from many types of cancer, including kidney cancer [39,40]. Compared to NHWs, U.S.-born HAs in California and Texas have 44–60% higher kidney cancer mortality rates, but foreign-born HAs had lower mortality rates than NHWs [6]. In Arizona, both foreign- and U.S.-born Mexican Americans had increased risk of mortality, but U.S.-born Mexican Americans had a greater risk. Although foreign-born HAs may have retained a healthier lifestyle while living in the U.S., foreign-born HAs are also more likely to be uninsured [14] and less likely to have access to healthcare [12,41] which may result in underestimation of the health data [42,43,44]. The difference in RCC mortality risk between U.S.-born and foreign-born Mexican Americans needs further investigation.

Our study suggests that treating HAs as a single group masks RCC burden among HAs in which some HA subgroups have a high RCC mortality rate. The literature has established the need for disaggregation of HA data by their origin and generational status to better assess the scope and magnitude of the cancer burden in this population [34,35]. Understanding the uniqueness of each HA subgroup including health behaviors, comorbidity, healthcare access, and genomic background is necessary to better understand the mechanisms contributing to advanced-stage RCC and high mortality.

The effects of neighborhood socioeconomic factors on mortality have been investigated for many cancer types [18,45,46,47]. Census-tract high school education, median household income, unemployment, and poverty rates are often used to assess associations between neighborhood characteristics and cancer outcomes. An emerging literature on structural inequality and neighborhood characteristics, such as redlining and segregation, have also been implicated in impacting cancer outcomes [19,20,21] strengthening the need to integrate census-tract level data to better understand patterns of cancer disparities. For example, the dominant role of poverty in cancer disparities is well-established [18,19,48]. Individuals living in neighborhoods with persistent poverty, defined as ≥20% of resident in poverty since 1980, face multiple challenges and have an increased cancer mortality risk [18].

A previous RCC study showed that including individual and neighborhood socioeconomic factors in the regression model eliminated survival disparities between NHWs and NHBs [10]. In studies of a single payer healthcare system, there was no evidence of disparities in survival between NHWs and NHBs [49], but HAs had higher risk of mortality compared to NHWs [17]. To our knowledge, this is the first study to explore risk factors of advanced-stage RCC and high mortality in AIs/ANs, and the results show that neighborhood socioeconomic factors may partially explain the RCC disparities in AIs/ANs, but not in HAs. Despite small sample sizes for AIs/ANs in both datasets, the introduction of neighborhood socioeconomic factors in the models attenuated the associations with advanced-stage RCC and mortality. The associations for HAs, particularly in Arizona, persisted after inclusion of neighborhood socioeconomic factors in the model. Among the factors evaluated in the study, high school education is of particular interest. High school graduation rate was inversely associated with advanced-stage RCC among Mexican Americans in NCDB and AIs/ANs in Arizona. Patients from neighborhoods with high socioeconomic disadvantage may have limited access to healthcare that delays diagnosis and treatment. Thus, they are more likely to present with advanced-stages and succumb to higher mortality.

We observed different association patterns in NCDB and ACR. The discrepancy may be due to case data ascertainment bias and high proportion of cases reported in large-scale hospitals in the NCDB [45]. Racial/ethnic minority groups and cases in western states, including Arizona, are underrepresented in the database [50,51]. HAs also tend to utilize smaller community-based hospitals rather than large-scale hospitals. As these smaller community-based hospitals would not report their cancer cases to the NCDB, the data would not be captured well in this database [14]. Because the study findings may not be generalizable to all racial/ethnic groups in the U.S., we carried out analysis using the ACR to assess RCC disparities in Arizona. ACR is a population-based registry that captures all cases in Arizona. AIs/ANs and HAs who were underrepresented in the NCDB were well-represented in the ACR. ACR also links AI/ANs case data to Indian Health Services reducing chances of misclassification, while NCDB does not link case information to Indian Health Services. NCDB data are often used to assess cancer care quality, but this study suggests a limitation of NCDB for cancer care quality assessment for racial/ethnic minority groups who are underrepresented in NCDB.

There are some limitations to the study. First, the data do not allow for a comprehensive analysis of socio-ecological factors, such as lifestyle (e.g., diet, physical activity, cigarette smoking), environmental exposure (e.g., occupational exposures and environmental toxin exposures), comorbidities (e.g., obesity, hypertension, and chronic kidney diseases), and genetics that have been previously discussed as RCC risk factors and common in racial/ethnic minority groups. The NCDB data contain the Charlson/Deyo Comorbidities Score, but specific disease information is not available. As a result, we were unable to fully assess if lifestyle factors and comorbidities affected the study findings. Second, geospatial data from Rural-Urban Continuum Codes were integrated into the analyses to provide information on the rural and urban setting of the individual RCC cases, but there was no data to better understand neighborhood characteristics (e.g., food desert and ethnic enclave) and to capture a nuanced snapshot of the individuals’ social context beyond the rural and urban categories. Additionally, geospatial data as they relate to healthcare systems and access were limited for this study. A better understanding of regional variations of healthcare systems and treatments available within these facilities, rurality or distance to tertiary referral centers, linkages of rural populations to care, and patient-related factors, would provide new dimensions for evaluating RCC outcomes and disparities.

## 5. Conclusions

Greater health disparities in RCC stage and mortality for HAs and RCC mortality for AIs/ANs were observed at the Arizona state level than the national level. A Hispanic paradox was observed at the national level but not at the Arizona state level. Impact of other RCC risk factors on RCC disparities in these populations need to be further investigated.

## Figures and Tables

**Table 1:** Logistic regression analysis results for advanced-stage.

Race/ethnicity	Unadjusted		Adjusted Model 1		Adjusted Model 2	
	OR (95%CI)	*p*	OR (95%CI)	*p*	OR (95%CI)	*p*
NCDB						
NHW vs. Other Groups						
Non-Hispanic White	Reference		Reference		Reference	
American Indian/Alaska Native	1.20 (1.08–1.33)	0.001	1.21 (1.06–1.38)	0.006	1.12 (0.98–1.29)	0.10
Non-Hispanic Black	0.68 (0.66–0.70)	<0.001	0.69 (0.67–0.72)	<0.001	0.69 (0.66–0.71)	<0.001
Asian American	1.01 (0.95–1.07)	0.76	0.96 (0.89–1.03)	0.22	0.98 (0.91–1.06)	0.65
Hispanic American	1.03 (1.00–1.05)	0.02	1.01 (0.98–1.04)	0.39	1.00 (0.97–1.04)	0.78
NHW vs. Hispanic American						
Non-Hispanic White	Reference		Reference		Reference	
Mexican/Chicano	1.35 (1.26–1.45)	<0.001	1.23 (1.12–1.35)	<0.001	1.22 (1.11–1.35)	<0.001
Puerto Rican	0.96 (0.82–1.12)	0.58	0.96 (0.78–1.18)	0.69	0.95 (0.77–1.17)	0.60
Cuban	1.21 (1.04–1.41)	0.01	1.08 (0.88–1.32)	0.49	1.07 (0.87–1.31)	0.55
South or Central American	1.07 (0.96–1.21)	0.23	1.02 (0.88–1.19)	0.76	1.04 (0.89–1.21)	0.63
Dominican	0.98 (0.75–1.29)	0.91	1.16 (0.81–1.67)	0.43	1.17 (0.81–1.71)	0.40
ACR						
NHW vs. Other Groups						
Non-Hispanic White	Reference		Reference		Reference	
American Indian/Alaska Native	1.29 (1.06–1.56)	0.01	1.42 (1.10–1.82)	0.006	1.23 (0.93–1.64)	0.15
Hispanic American	1.15 (1.01–1.31)	0.04	1.12 (0.94–1.33)	0.20	1.08 (0.90–1.29)	0.42
NHW vs. Mexican American						
Non-Hispanic White	Reference				Reference	
Mexican American	2.24 (1.89–2.67)	<0.001	2.10 (1.66–2.64)	<0.001	2.02 (1.58–2.58)	<0.001
NHW vs. U.S.- and Foreign-Born Mexican American						
Non-Hispanic White	Reference		Reference		Reference	
U.S.-Born Mexican American	2.67 (2.09–3.41)	<0.001	2.33 (1.68–3.24)	<0.001	2.27 (1.62–3.18)	<0.001
Foreign-Born Mexican American	2.25 (1.68–3.00)	<0.001	2.15 (1.47–3.13)	<0.001	2.05 (1.39–3.03)	<0.001

ACR, Arizona Caner Registry; NCDB, National Cancer Database; NHW, Non-Hispanic White.

**Table 2:** Cox Regression analysis for all-cause mortality in NCDB.

Race/ethnicity	Unadjusted		Adjusted Model 1		Adjusted Model 2	
	HR (95%CI)	*p*	HR (95%CI)	*p*	HR (95%CI)	*p*
NHW vs. Other Groups						
Non-Hispanic White	Reference		Reference		Reference	
American Indian/Alaska Native	1.10 (1.01–1.20)	0.04	1.12 (0.97–1.30)	0.13	1.04 (0.90–1.21)	0.58
Non-Hispanic Black	1.00 (0.98–1.02)	0.81	1.12 (1.08–1.16)	<0.001	1.05 (1.02–1.09)	0.003
Asian American	0.83 (0.79–0.87)	<0.001	0.84 (0.77–0.91)	<0.001	0.73 (0.63–0.84)	<0.001
Hispanic American	0.91 (0.89–0.93)	<0.001	0.93 (0.90–0.96)	<0.001	0.91 (0.88–0.94)	<0.001
NHW vs. Hispanic American						
Non-Hispanic White	Reference		Reference		Reference	
Mexican/Chicano	0.99 (0.93–1.05)	0.67	0.95 (0.86–1.06)	0.37	0.91 (0.82–1.01)	0.06
Puerto Rican	0.80 (0.70–0.93)	0.003	0.83 (0.65–1.05)	0.12	0.78 (0.61–0.99)	0.046
Cuban	1.14 (1.01–1.29)	0.03	1.03 (0.85–1.24)	0.79	0.97 (0.80–1.17)	0.73
South or Central American	0.62 (0.55–0.71)	<0.001	0.73 (0.60–0.89)	0.002	0.72 (0.59–0.88)	0.001
Dominican	0.75 (0.58–0.98)	0.03	0.84 (0.57–1.24)	0.39	0.79 (0.53–1.17)	0.24

NHW, Non-Hispanic White.

**Table 3:** All-cause and RCC-specific mortality in ACR.

Race/ethnicity	Unadjusted		Adjusted Model 1		Adjusted Model 2	
	HR (95%CI)	*p*	HR (95%CI)	*p*	HR (95%CI)	*p*
All-cause mortality						
NHW vs. Other Groups						
Non-Hispanic White	Reference		Reference		Reference	
American Indian/Alaska Native	1.20 (1.05–1.37)	0.009	1.77 (1.40–2.24)	<0.001	1.33 (1.03–1.72)	0.03
Hispanic American	1.11 (1.01–1.22)	0.03	1.37 (1.17–1.60)	<0.001	1.16 (0.98–1.38)	0.08
NHW vs. Mexican American	34					
Non-Hispanic White	Reference		Reference		Reference	
Mexican American	2.46 (2.23–2.72)	<0.001	2.86 (2.41–3.39)	<0.001	2.30 (1.91–2.77)	<0.001
NHW vs. U.S.- and Foreign-Born Mexican American						
Non-Hispanic White	Reference		Reference		Reference	
U.S.-Born Mexican American	3.16 (2.80–3.57)	<0.001	3.81 (3.10–4.68)	<0.001	3.21 (2.61–3.98)	<0.001
Foreign-Born Mexican American	1.95 (1.62–2.34)	<0.001	1.91 (1.39–2.61)	<0.001	1.50 (1.09–2.06)	0.01
RCC-specific mortality						
Race/ethnicity						
Non-Hispanic White	Reference		Reference		Reference	
American Indian/Alaska Native	1.12 (0.85–1.48)	0.42	1.20 (0.85–1.71)	0.30	1.09 (0.72–1.65)	0.68
Hispanic American	1.17 (0.97–1.41)	0.11	1.05 (0.81–1.37)	0.72	1.01 (0.76–1.33)	0.96
NHW vs. Mexican American						
Non-Hispanic White	Reference		Reference		Reference	
Mexican American	2.72 (2.24–3.29)	<0.001	1.98 (1.49–2.63)	<0.001	1.76 (1.30–2.38)	<0.001
NHW vs. U.S.- and Foreign-Born Mexican American						
Non-Hispanic White	Reference		Reference		Reference	
U.S.-Born Mexican American	3.63 (2.89–4.54)	<0.001	3.06 (2.27–4.14)	<0.001	2.79 (2.05–3.81)	<0.001
Foreign-Born Mexican American	1.77 (1.21–2.61)	<0.01	0.83 (0.45–1.53)	0.54	0.71 (0.37–1.35)	0.29

NHW, Non-Hispanic White; RCC, renal cell carcinoma.

## Data Availability

The data used for current study are available from the NCDB (https://www.facs.org/Quality-Programs/Cancer/NCDB) and ACR (https://www.azdhs.gov/preparedness/public-health-statistics/cancer-registry/index.php).

## Supplementary Materials

The following are available online at https://www.mdpi.com/2072-6694/13/5/990/s1, Table S1: Characteristics of patients across racial/ethnic groups in NCDB (*n* = 405,073), Table S2: Characteristics of non-Hispanic Whites, American Indian/Alaska Native, and Hispanic Americans in ACR (*n* = 9337), Table S3: Factors associated with advanced-stage in American Indians/Alaskan Natives in NCDB (*n* = 1811), Table S4: Factors associated with advanced stage in Mexican Americans in NCDB (*n* = 3745), Table S5: Factors associated with advanced-stage in American Indians/Alaskan Natives in ACR (*n* = 632), Table S6: Factors associated with advanced-stage diagnosis in Mexican Americans in ACR (*n* = 739), Table S7: Cox Regression analysis for all-cause mortality in NCDB stratified based on TNM stage (early vs. late), Table S8: Cox Regression analysis for all-cause mortality in ACR stratified based on TNM stage (early vs. late), Table S9: RCC-specific mortality in ACR stratified based on TNM stage (early vs. late).
