# Supplementary material for: Renal Cell Carcinoma Health Disparities in Stage and Mortality among American Indians/Alaska Natives and Hispanic Americans: Comparison of National Cancer Database and Arizona Cancer Registry Data
Source: Cancers (Basel). 2021 Feb 27;13(5):990. doi: 10.3390/cancers13050990 (PMC7956712; doi:10.3390/cancers13050990)
Supplement: Supplementary file 1 [file cancers-13-00990-s001.pdf]

Supplementary Materials

Renal Cell Carcinoma Health Disparities in Stage and Mortality among American Indians/Alaska Natives and Hispanic Americans: Comparison of National Cancer Database and Arizona Cancer Registry Data

Celina I. Valencia, Samer Asmar, Chiu-Hsieh Hsu, Francine C. Gachupin, Ava C. Wong, Juan Chipollini, Benjamin R. Lee and Ken Batai

Supplementary Methods

Separate analyses for American Indians/Alaska Natives (AIs/ANs) and Mexican Americans were performed in each dataset, National Cancer Database (NCDB) and Arizona Cancer Registry (ACR) to identify specific factors associated with advanced-stage renal cell carcinoma (RCC). For each dataset and each racial/ethnic group, bivariate logistic regression was performed for each variable, including patients' demographic, neighborhood characteristics, residence pattern, and health care access factors. Variables that were significantly associated with advanced-stage RCC were included in the adjusted model. For Arizona ACR, percent Native language (<4% vs. ≥4%) and percent Spanish language (<10%, ≥10%, <25%, ≥25%, <50%, or ≥50%) use were obtained from Arizona United States census data to assess if patients from neighborhood with high Native or Spanish language use have more disadvantage. For AIs/ANs in Arizona, percent high school graduation rate was categorized into two (≥70% vs. <70%), since not many AIs/ANs patients came from neighborhood with ≥90% high school graduation rate.

Table S1. Characteristics of patients across racial/ethnic groups in NCDB (*n* = 405,073).

Characteristics	NHW	AI/AN	NHB	AsianA	HA					
	(*n* = 302,230)	(*n* = 1811)	(*n* = 45,334)	(*n* = 6390)	All HA (*n* = 49,308)	Mexican/Chicano (*n* = 3745)	Puerto Rican (*n* = 866)	Cuban (*n* = 798)	South or Central American (*n* = 1464)	Dominican (*n* = 269)
Age, mean (SD)	63.2 (12.7)	58.5 (12.1)	60.4 (12.4)	61.4 (13.6)	60.4 (13.4)	59.0 (13.2)	60.0 (13.2)	63.6 (12.7)	58.3 (13.0)	60.4 (14.0)
Gender, *n* (%)										
Male	190,841 (63.1)	1039 (57.4)	26,776 (59.1)	4175 (65.3)	29,785 (60.4)	2264 (60.5)	505 (58.3)	518 (64.9)	875 (59.8)	164 (61.0)
Female	111,389 (36.9)	772 (42.6)	18,558 (40.9)	2215 (34.7)	19,523 (39.6)	1481 (39.5)	361 (41.7)	280 (35.1)	589 (40.2)	105 (39.0)
Grade, *n* (%)										
1&2	140,469 (64.7)	920 (68.8)	19,878 (65.6)	2808 (62.5)	23,957 (67.4)	1683 (64.6)	403 (67.2)	355 (67.1)	711 (69.0)	108 (65.9)
3&4	76,664 (35.3)	417 (31.2)	10,410 (34.4)	1682 (37.5)	11,569 (32.6)	922 (35.4)	197 (32.8)	174 (32.9)	320 (31.0)	56 (34.1)
Stage, *n* (%)										
I&II	201,416 (72.3)	1153 (68.5)	32,897 (79.4)	4275 (72.1)	32,559 (71.7)	2295 (65.9)	596 (73.1)	516 (68.3)	975 (70.8)	188 (72.6)
III&IV	77,325 (27.7)	529 (31.5)	8559 (20.6)	1656 (27.9)	12,832 (28.6)	1187 (34.1)	219 (26.9)	240 (31.7)	402 (29.2)	71 (27.4)

Patients from other racial/ethnic groups and unknown race/ethnicity are not shown. Among HAs, patients with known national/geographic origin were shown on the table. Patients with unknown RCC histological subtype were not included.
[a] Not Otherwise Specified (NOS).

Table S2. Characteristics of non-Hispanic Whites, American Indian/Alaska Native, and Hispanic Americans in ACR (*n* = 9337).

Characteristics	NHW (*n* = 6965)	AI/AN (*n* = 632)	HA			
			All HA (*n* = 1740)	Mexican American (*n* = 739)	US-Born Mexican American (*n* = 335)	Mexico-Born Mexican American (*n* = 238)
Age, mean (SD)	64.3 (12.6)	58.9 (12.9)	59.3 (13.2)	62.0 (13.2)	63.5 (13.5)	61.7 (12.3)
Gender, *n* (%)						
Male	4525 (65.0)	401 (63.4)	1052 (60.5)	464 (62.8)	228 (68.1)	141 (59.2)
Female	2440 (35.0)	231 (36.6)	688 (39.5)	275 (37.2)	107 (31.9)	97 (40.8)
Grade, *n* (%)						
1&2	3170 (64.9)	211 (70.6)	816 (67.0)	211 (59.8)	116 (58.3)	95 (61.7)
3&4	1714 (35.1)	88 (29.4)	402 (33.0)	142 (40.2)	83 (41.7)	59 (38.3)
Stage, *n* (%)						
I&II	4174 (73.6)	345 (68.5)	959 (70.8)	246 (52.8)	138 (50.9)	108 (55.4)
III&IV	1497 (26.4)	163 (31.5)	495 (29.2)	220 (47.2)	133 (49.1)	87 (44.6)

Patients from other racial/ethnic groups and unknown race/ethnicity are not shown.

Table S3. Factors associated with advanced-stage in American Indians/Alaskan Natives in NCDB (*n* = 1811).

Characteristics	Unadjusted			Adjusted	
	n	OR (95% C.I.)	*p*	OR (95% C.I.)	*p*
Age			<0.001		<0.001
18–49	419 (23.1)	Reference		Reference	
50–59	533 (29.4)	2.11 (1.55–2.88)		2.18 (1.46–3.26)	
60–69	502 (27.7)	2.21 (1.61–3.02)		2.41 (1.61–3.62)	
70–79	294 (16.2)	2.31 (1.63–3.28)		2.86 (1.81–4.53)	
80 or older	63 (3.5)	2.35 (1.27–4.25)		1.75 (0.64–4.79)	
Gender			0.001		
Male	1039 (57.4)	Reference		Reference	0.002
Female	772 (42.6)	0.69 (0.56–0.85)		0.64 (0.48–0.84)	
Histologic Subtype			<0.001		<0.001
Clear Cell	1077 (86.3)	Reference		Reference	
Papillary	68 (5.4)	0.63 (0.33–1.19)		0.53 (0.26–1.07)	
Chromophobe	41 (3.3)	0.63 (0.28–1.46)		0.68 (0.29–1.60)	
Others	62 (5.0)	3.02 (1.76–5.17)		2.94 (1.68–5.17)	
Facility Type			0.14		
Comprehensive Community Cancer Program	774 (45.5)	Reference			
Community Cancer Program	214 (12.6)	1.31 (0.95–1.83)			
Integrated Network Cancer Program	105 (6.2)	0.89 (0.56–1.40)			
Academic/Research Program	607 (35.7)	1.23 (0.98–1.56)			
Insurance Type			0.28		
Private	559 (30.9)	Reference			
Public (Medicare/Medicaid/Others)	1113 (61.5)	1.14 (0.90–1.43)			
Not insured	65 (3.6)	1.24 (0.70–2.20)			
Not known	74 (4.1)	1.64 (0.97–2.77)			
Metro, urban vs. rural area			0.10		0.14
Metro	867 (48.7)	Reference		Reference	
Urban	737 (41.4)	1.28 (1.02–1.59)		1.04 (0.76–1.43)	
Rural	176 (9.9)	1.15 (0.80–1.65)		0.70 (0.41–1.21)	
Great Circle Distance, mean (SD)	82.2 (160.3)	1.01 (1.00–1.02)	0.02	1.01 (1.003–1.02)	0.008
Median Income			0.03		0.72
<$38,000	799 (44.6)	Reference		Reference	
$38,000–$47,999	460 (25.7)	0.75 (0.58–0.97)		0.98 (0.68–1.39)	
$48,000–62,999	347 (19.4)	0.80 (0.60–1.06)		0.81 (0.52–1.28)	
$63,000+	185 (10.3)	0.63 (0.44–0.92)		0.73 (0.38–1.39)	
% no high school degree			0.03		
≥21%	594 (33.1)	References		Reference	

13.0–20.9%	573 (32.0)	0.73 (0.57–0.94)		0.89 (0.63–1.25)	
7.0–12.9%	447 (24.9)	0.72 (0.54–0.94)		0.80 (0.53–1.22)	
<7.0%	179 (10.0)	0.67 (0.45–0.98)		1.14 (0.59–2.18)	
Charlson/Deyo Score			0.87		
0	1139 (62.9)	Reference			
1	469 (25.9)	0.96 (0.76–1.23)			
2	203 (11.2)	0.92 (0.66–1.28)			
Diagnosis Year			0.03		0.81
2004–2006	258 (14.2)	Reference		Reference	
2007–2009	414 (22.9)	0.65 (0.46–0.91)		0.82 (0.50–1.37)	
2010–2012	515 (28.4)	0.66 (0.42–0.91)		0.85 (0.53–1.38)	
2013–2015	624 (34.5)	0.63 (0.45–0.86)		0.78 (0.49–1.24)	

Table S4. Factors associated with advanced-stage in Mexican Americans in NCDB (n = 3745).

**Characteristics**	**Unadjusted**			**Adjusted**	
	n	**OR (95% C.I.)**	***p***	**OR (95% C.I.)**	***p***
Age			<0.001		0.03
18–49	897 (24.0)	Reference		Reference	
50–59	1014 (27.1)	1.50 (1.22–1.83)		1.20 (0.90–1.61)	
60–69	978 (26.1)	1.71 (1.40–2.10)		1.53 (1.15–2.05)	
70–79	630 (16.8)	1.76 (1.40–2.21)		1.57 (1.13–2.20)	
80 or older	226 (6.0)	1.52 (1.09–2.11)		1.32 (0.79–2.19)	
Gender			<0.001		
Male	2264 (60.5)	Reference		Reference	<0.001
Female	1481 (39.5)	0.74 (0.64–0.85)		0.69 (0.57–0.84)	
Histologic Subtype			<0.001		<0.001
Clear Cell	2126 (83.5)	Reference		Reference	
Papillary	153 (6.0)	0.47 (0.29–0.74)		0.27 (0.22–0.61)	
Chromophobe	145 (3.9)	0.32 (0.19–0.54)		0.29 (0.16–0.53)	
Others	122 (3.3)	4.12 (2.77–6.11)		4.48 (2.85–7.05)	
Facility Type			0.001		0.10
Comprehensive Community Cancer Program	1374 (39.7)	Reference		Reference	
Community Cancer Program	363 (10.5)	1.24 (0.97–1.60)		1.35 (0.97–1.88)	
Integrated Network Cancer Program	131 (3.8)	0.88 (0.58–1.33)		0.90 (0.51–1.59)	
Academic/Research Program	1590 (46.0)	1.36 (1.16–1.60)		1.26 (1.02–1.56)	
Insurance Type			<0.001		0.14
Private	1269 (33.9)	Reference		Reference	
Public (Medicare/Medicaid/Others)	1900 (50.7)	1.41 (1.20–1.66)		1.10 (0.88–1.38)	
Not insured	459 (12.3)	1.91 (1.52–2.40)		1.49 (1.06–2.10)	
Not known	117 (3.1)	1.51 (1.01–2.27)		1.32 (0.68–2.57)	
Metro, urban vs. rural area			0.33		
Metro	3411 (93.1)	Reference			
Urban	235 (6.3)	0.82 (0.60–1.10)			
Rural	17 (0.5)	1.43 (0.50–4.13)			
Great Circle Distance	23.9 (71.2)	1.00 (0.99–1.01)	0.78		
Median Income			0.01		0.06
<$38,000	888 (23.9)	Reference		Reference	
$38,000–$47,999	963 (25.9)	1.04 (0.86–1.27)		1.30 (0.99–1.69)	
$48,000–62,999	1112 (29.9)	0.82 (0.68–0.99)		0.98 (0.74–1.29)	
$63,000+	758 (20.4)	0.80 (0.64–0.98)		1.33 (0.92–1.92)	
% no high school degree			<0.001		0.01
≥21%	2137 (57.4)	References		Reference	
13.0–20.9%	735 (19.7)	0.80 (0.67–0.97)		0.82 (0.63–1.07)	
7.0–12.9%	565 (15.2)	0.64 (0.52–0.80)		0.58 (0.42–0.82)	
<7.0%	285 (7.7)	0.65 (0.49–0.87)		0.59 (0.37–0.94)	
Charlson/Deyo Score			0.33		
0	2798 (74.7)	Reference			
1	697 (18.6)	0.88 (0.73–1.06)			
2	250 (6.7)	0.90 (0.67–1.19)			
Diagnosis Year			0.10		0.27
2004–2006	608 (16.2)	Reference		Reference	
2007–2009	910 (24.3)	0.89 (0.71–1.12)		0.87 (0.63–1.19)	
2010–2012	1100 (29.4)	0.77 (0.62–0.96)		0.76 (0.56–1.03)	

2013–2015	1127 (30.1)	0.82 (0.66–1.02)		0.92 (0.68–1.24)	

Table S5. Factors associated with advanced-stage in American Indians/Alaskan Natives in ACR (n = 632).

**Characteristics**	**Unadjusted**			**Adjusted**	
	n **(%)**	**OR (95% C.I.)**	p	**OR (95% C.I.)**	p
Age			0.10		0.06
20–49	146 (23.1)	Reference		Reference	
50–65	297 (47.1)	1.60 (0.99–2.27)		2.08 (1.14–3.79)	
66–79	151 (23.9)	0.96 (0.54–1.71)		1.07 (0.50–2.28)	
80+	37 (5.9)	1.55 (0.65–3.69)		1.69 (0.37–7.70)	
Gender			0.009		0.03
Male	401 (63.4)	Reference		Reference	
Female	231 (36.6)	0.58 (0.39–0.87)		0.55 (0.32–0.95)	
Histologic Subtype			<0.001		<0.001
Clear Cell	377 (88.7)	Reference		Reference	
Papillary	22 (5.2)	1.20 (0.44–3.27)		1.26 (0.43–3.69)	
Chromophobe	8 (1.9)	0.44 (0.05–2.66)		0.52 (0.06–4.64)	
Others	18 (4.2)	2.61 (0.89–7.66)		3.73 (1.12–12.48)	
Marital Status			0.27		
Married	283 (44.8)	Reference			
Single	181 (28.6)	0.87 (0.56–1.35)			
Separated/divorced/widowed	107 (16.9)	0.78 (0.46–1.32)			
Not known	61 (9.7)	0.47 (0.22–1.03)			
% Native language			0.84		
<4%	242 (38.3)	Reference			
≥4%	388 (61.4)	0.96 (0.66–1.41)			
RUCC 2013			0.52		
1 or 2	304 (48.1)	Reference			
3–7	327 (51.7)	1.13 (0.78–1.64)			
% high school degree graduation			0.77		0.04
≥70%	215 (34.0)	Reference			
<70%	415 (65.7)	1.14 (0.76–1.69)		2.73 (1.04–7.17)	
% unemployment			0.96		
<5%	140 (22.2)	References			
≥5, <10%	147 (23.3)	1.07 (0.62–1.86)			
≥10%	343 (54.3)	1.06 (0.66–1.70)			
% Poverty Rate			0.10		0.08
<10%	103 (16.3)	Reference		Reference	
≥10, <20%	113 (17.9)	1.65 (0.88–3.09)		0.98 (0.41–2.35)	
≥20%	414 (65.5)	0.94 (0.58–1.65)		0.37 (0.12–1.15)	
Diagnosis Year			0.09		0.048
2007–2008	108 (17.1)	Reference		Reference	
2009–2010	121 (19.1)	1.25 (0.67–2.32)		1.73 (0.73–4.12)	
2011–2012	134 (21.2)	0.86 (0.52–1.79)		1.09 (0.46–2.60)	
2013–2014	121 (19.1)	1.96 (1.08–3.56)		2.89 (1.28–6.49)	
2015–2016	148 (23.4)	1.40 (0.72–2.69)		1.90 (0.80–4.48)	

Table S6. Factors associated with advanced-stage diagnosis in Mexican Americans in ACR (n = 739).

Characteristics	Unadjusted			Adjusted	
	n	OR (95% C.I.)	*p*	OR (95% C.I.)	*p*
Age			0.95		0.69
20–49	139 (18.9)	Reference		Reference	
50–65	295 (40.1)	1.14 (0.73–1.78)		1.15 (0.55–2.39)	
66–79	239 (32.5)	1.11 (0.69–1.77)		1.13 (0.53–2.44)	
80+	62 (8.4)	1.03 (0.49–2.17)		2.20 (0.60–8.07)	
Gender			0.006		0.054
Male	464 (62.8)	Reference		Reference	
Female	275 (37.2)	0.62 (0.44–0.87)		0.56 (0.32–1.01)	
Country of Origin			0.84		0.47
U.S.-Born	335 (58.5)	Reference		Reference	
Mexico-Born	238 (41.5)	0.84 (0.58–1.22)		0.83 (0.49–1.41)	
Histologic Subtype			0.37		0.19
Clear Cell	363 (82.5)	Reference		Reference	
Papillary	30 (6.8)	1.11 (0.49–2.54)		1.14 (0.45–2.86)	
Chromophobe	25 (5.7)	1.53 (0.50–4.66)		1.84 (0.36–9.51)	
Others	22 (5.0)	2.23 (0.85–5.81)		3.12 (1.08–9.06)	
Marital Status			0.23		0.06
Married	390 (52.8)	Reference		Reference	
Single	147 (19.9)	0.97 (0.63–1.48)		0.84 (0.42–1.72)	
Separated/divorced/widowed	166 (22.5)	0.76 (0.50–1.14)		0.51 (0.26–1.02)	
Not known	36 (4.6)	1.93 (0.78–4.79)		3.83 (0.81–18.18)	
% Spanish language			0.80		
<10%	297 (17.1)	Reference			
≥10, <25%	448 (28.0)	0.86 (0.60–1.24)			
≥25, <50%	393 (22.6)	0.88 (0.61–1.27)			
≥50%	561 (32.3)	0.98 (0.69–1.38)			
RUCC 2013			0.03		0.50
1 or 2	1332 (76.6)	Reference		Reference	
3–7	407 (23.4)	1.36 (1.04–1.79)		1.22 (0.68–2.21)	
% no high school degree			0.83		
≥90%	238 (13.7)	Reference			
≥70, <90%	725 (41.7)	1.05 (0.73–1.52)			
<70%	776 (44.6)	0.97 (0.68–1.40)			
% unemployment			0.40		
<5%	587 (33.8)	References			
≥5%, <10%	821 (47.2)	1.10 (0.84–1.43)			
≥10%	331 (19.0)	1.26 (0.90–1.75)			
% Poverty Rate			0.12		0.61
<10%	473 (27.2)	Reference		Reference	
≥10%, <20%	580 (33.4)	1.38 (1.02–1.88)		0.81 (0.42–1.54)	
≥20%	686 (39.4)	1.19 (0.88–1.60)		0.71 (0.38–1.38)	
Diagnosis Year			0.01		0.003
2007–2008	315 (18.1)	Reference		Reference	
2009–2010	344 (19.8)	1.17 (0.81–1.71)		1.08 (0.47–2.50)	
2011–2012	359 (20.6)	0.99 (0.68–1.45)		1.15 (0.50–2.64)	
2013–2014	335 (19.3)	1.24 (0.85–1.80)		2.70 (1.17–6.27)	
2015–2016	387 (22.2)	1.74 (1.17–2.61)		3.97 (1.62–9.73)	

Table S7. Cox Regression analysis for all-cause mortality in NCDB stratified based on TNM stage (early vs. late).

Race/Ethnicity	Unadjusted		Adjusted Model 1		Adjusted Model 2	
	**HR (95%CI)**	***P***	**HR (95%CI)**	***p***	**HR (95%CI)**	***p***
NHW vs. Other Groups						
Early-Stage						
Non-Hispanic White	Reference		Reference		Reference	
American Indian/Alaska Native	1.01 (0.88–1.17)	0.57	1.13 (0.92–1.38)	0.25	1.04 (0.84–1.27)	0.74
Non-Hispanic Black	1.12 (1.09–1.15)	<0.001	1.12 (1.13–1.23)	<0.001	1.09 (1.04–1.13)	<0.001
Asian American	0.67 (0.61–0.73)	<0.001	0.72 (0.64–0.82)	<0.001	0.76 (0.67–0.86)	<0.001
Hispanic American	0.85 (0.82–0.87)	<0.001	0.90 (0.86–0.94)	<0.001	0.87 (0.84–0.91)	<0.001
Late-Stage						
Non-Hispanic White	Reference		Reference		Reference	
American Indian/Alaska Native	1.07 (0.95–1.20)	0.29	1.07 (0.87–1.32)	0.52	1.01 (0.81–1.26)	0.93
Non-Hispanic Black	1.22 (1.18–1.25)	<0.001	1.09 (1.04–1.16)	0.002	1.06 (0.99–1.12)	0.06
Asian American	0.94 (0.88–1.01)	0.10	0.94 (0.87–1.32)	0.25	0.97 (0.87–1.09)	0.61
Hispanic American	0.95 (0.92–0.97)	<0.001	0.97 (0.93–1.01)	0.12	0.96 (0.91–0.99)	0.04
NHW vs. Hispanic American						
Early Stage						
Non-Hispanic White	Reference		Reference		Reference	
Mexican/Chicano	0.80 (0.72–0.90)	<0.001	0.95 (0.82–1.11)	0.53	0.89 (0.77–1.04)	0.14
Puerto Rican	0.67 (0.53–0.85)	0.001	0.61 (0.42–0.87)	0.007	0.56 (0.39–0.81)	0.002
Cuban	0.83 (0.67–1.03)	0.10	0.81 (0.60–1.10)	0.17	0.75 (0.56–1.02)	0.07
South or Central American	0.56 (0.46–0.69)	<0.001	0.71 (0.53–0.94)	0.02	0.70 (0.53–0.93)	0.01
Dominican	0.74 (0.49–1.12)	0.15	0.83 (0.46–1.50)	0.53	0.77 (0.43–1.40)	0.39
Late Stage						
Non-Hispanic White	Reference		Reference		Reference	
Mexican/Chicano	0.95 (0.87–1.03)	0.19	0.94 (0.82–1.08)	0.37	0.90 (0.78–1.04)	0.16
Puerto Rican	0.95 (0.79–1.15)	0.61	1.09 (0.79–1.50)	0.60	1.07 (0.77–1.48)	0.69
Cuban	1.33 (1.14–1.55)	<0.001	1.22 (0.95–1.56)	0.12	1.16 (0.91–1.50)	0.23
South or Central American	0.67 (0.57–0.79)	<0.001	0.76 (0.58–1.01)	0.054	0.75 (0.57–0.99)	0.047
Dominican	0.81 (0.58–1.14)	0.22	0.94 (0.56–1.59)	0.81	0.89 (0.53–1.51)	0.67

Model 1: adjusted for age category, gender, RCC histologic subtype, grade (1 and 2 vs. 3 and 4), facility type, insurance type, Charlson/Deyo Score, and year of diagnosis. Model 2: adjusted for age category, gender, RCC histologic subtype, grade (1 and 2 vs. 3 and 4), facility type, insurance type, great circle distance, neighborhood characteristics (median income and % high school graduation), Charlson/Deyo Score, and year of diagnosis. Unadjusted model included all RCC histologic subtypes even if subtype was not specified (NOS), while adjusted model excluded NOS RCC subtype.

Table S8. Cox Regression analysis for all-cause mortality in ACR stratified based on TNM stage (early vs. late).

Race/Ethnicity	Unadjusted		Adjusted Model 1		Adjusted Model 2	
	HR (95%CI)	*P*	HR (95%CI)	*p*	HR (95%CI)	*p*
NHW vs. Other Groups						
Early-Stage						
Non-Hispanic White	Reference		Reference		Reference	
American Indian/Alaska Native	1.36 (1.10–1.67)	0.004	1.74 (1.29–2.36)	<0.001	1.33 (0.94–1.89)	0.11
Hispanic American	1.05 (0.90–1.22)	0.57	1.23 (0.99–1.52)	0.06	1.01 (0.80–1.27)	0.96
Late-Stage						
Non-Hispanic White	Reference		Reference		Reference	
American Indian/Alaska Native	1.14 (0.93–1.39)	0.22	1.51 (1.12–1.84)	0.03	1.29 (0.84–1.96)	0.24
Hispanic American	1.17 (1.02–1.34)	0.03	1.43 (1.12–1.83)	0.005	1.33 (1.02–1.74)	0.04
NHW vs. Mexican American	34					
Early-Stage						
Non-Hispanic White	Reference		Reference		Reference	
Mexican American	2.65 (2.24–3.13)	<0.001	2.78 (2.20–3.51)	<0.001	2.25 (1.72–2.94)	<0.001
Late-Stage						
Non-Hispanic White	Reference		Reference		Reference	
Mexican American	1.72 (1.49–1.99)	<0.001	2.53 (1.94–3.31)	<0.001	2.19 (1.64–2.93)	<0.001
NHW vs. U.S.- and Foreign-Born Mexican American						
Early-Stage						
Non-Hispanic White	Reference		Reference		Reference	
U.S.-Born Mexican American	3.68 (3.02–4.48)	<0.001	3.62 (2.77–4.72)	<0.001	3.07 (2.27–4.14)	<0.001
Foreign-Born Mexican American	1.67 (1.18–2.36)	0.004	1.54 (0.93–2.55)	0.09	1.23 (0.73–2.09)	0.44
Late-Stage						
Non-Hispanic White	Reference		Reference		Reference	
U.S.-Born Mexican American	2.01 (1.67–2.43)	<0.001	3.13 (2.21–4.44)	<0.001	2.76 (1.92–3.97)	<0.001
Foreign-Born Mexican American	1.38 (1.09–1.77)	0.01	2.04 (1.35–3.06)	0.001	1.67 (1.08–2.58)	0.02

Model 1: Adjusted for age category, gender, marital status, RCC subtypes (excluding NOS), grade (1/2 vs. 3/4), and diagnosis year (categorical). Model 2: Adjusted for age category, gender, marital status, RCC subtypes (excluding NOS), stage (I/II vs. III/IV), grade (1/2 vs. 3/4), diagnosis year (categorical), percent high school graduation rate (≥90%, 70–90%, and <70%), percent unemployment (<5%. 5–10%, and ≥10%), and percent poverty (<10%, 10–20%, and ≥20%). Unadjusted model included all RCC histologic subtypes even if subtype was not specified (NOS), while adjusted model excluded NOS RCC subtype.

Table S9. RCC-specific mortality in ACR stratified based on TNM stage (early vs. late).

Race/ethnicity	Unadjusted		Adjusted Model 1		Adjusted Model 2	
	HR (95%CI)	*P*	HR (95%CI)	*p*	HR (95%CI)	*p*
NHW vs. Other Groups						
Early-Stage						
Non-Hispanic White	Reference		Reference		Reference	
American Indian/Alaska Native	1.10 (0.62–1.97)	0.74	0.96 (0.47–1.94)	0.96	1.01 (0.41–2.46)	0.99
Hispanic American	1.01 (0.67–1.52)	0.98	0.97 (0.61–1.55)	0.97	0.90 (0.57–1.43)	0.66
Late-Stage						
Non-Hispanic White	Reference		Reference		Reference	
American Indian/Alaska Native	1.04 (0.75–1.44)	0.80	1.21 (0.80–1.83)	0.36	1.07 (0.66–1.73)	0.80
Hispanic American	1.10 (0.87–1.40)	0.43	1.03 (0.74–1.42)	0.87	1.00 (0.71–1.42)	0.99
NHW vs. Mexican American						
Early-Stage						
Non-Hispanic White	Reference		Reference		Reference	
Mexican American	2.63 (1.73–3.99)	<0.0001	2.30 (1.44–3.67)	<0.001	2.08 (1.26–3.43)	<0.01
Late-Stage						
Non-Hispanic White	Reference		Reference		Reference	
Mexican American	1.76 (1.38–2.24)	<0.0001	1.71 (1.20–2.43)	<0.01	1.51 (1.04–2.18)	0.03
NHW vs. U.S.- and Foreign-Born Mexican American						
Early-Stage						
Non-Hispanic White	Reference		Reference		Reference	
U.S.-Born Mexican American	3.73 (2.37–5.87)	<0.0001	3.03 (1.81–5.07)	<0.0001	2.84 (1.62–4.98)	<0.001
Foreign-Born Mexican American	0.32 (0.05, 2.33)	0.26	0.34 (0.05–2.50)	0.29	0.34 (0.05–2.55)	0.30
Late-Stage						
Non-Hispanic White	Reference		Reference		Reference	
U.S.-Born Mexican American	2.48 (1.87–3.28)	<0.0001	2.69 (1.88–3.85)	<0.0001	2.43 (1.70–3.48)	<0.0001
Foreign-Born Mexican American	1.08 (0.68–1.73)	0.74	0.90 (0.45–1.79)	0.76	0.74 (0.36–1.54)	0.42

Model 1: Adjusted for age category, gender, marital status, RCC subtypes, stage (I/II vs. III/IV) grade (1/2 vs. 3/4), and diagnosis year (categorical). Model 2: Adjusted for age category, gender, marital status, RCC subtypes, stage (I/II vs. III/IV) grade (1/2 vs. 3/4), diagnosis year (categorical), percent high school graduation rate (≥90%, 70–90%, and <70%), percent unemployment (<5%. 5–10%, and ≥10%), and percent poverty (<10%, 10–20%, and ≥20%).
